# Gut Microbiota Signatures Predict Host and Microbiota Responses to Dietary Interventions in Obese Individuals

**DOI:** 10.1371/journal.pone.0090702

**Published:** 2014-03-06

**Authors:** Katri Korpela, Harry J. Flint, Alexandra M. Johnstone, Jenni Lappi, Kaisa Poutanen, Evelyne Dewulf, Nathalie Delzenne, Willem M. de Vos, Anne Salonen

**Affiliations:** 1 Immunobiology Research Program, Department of Bacteriology and Immunology, University of Helsinki, Helsinki, Finland; 2 Rowett Institute of Nutrition and Health, University of Aberdeen, Aberdeen, United Kingdom; 3 Department of Clinical Nutrition, University of Eastern Finland, Kuopio, Finland; 4 VTT Technical Research Centre, Espoo and Kuopio, Finland; 5 Louvain Drug Research Institute, Catholic University of Louvain, Bruxelles, Belgium; 6 Department of Veterinary Biosciences, University of Helsinki, Helsinki, Finland; 7 Laboratory of Microbiology, Wageningen University, Wageningen, the Netherlands; Charité-University Medicine Berlin, Germany

## Abstract

**Background:**

Interactions between the diet and intestinal microbiota play a role in health and disease, including obesity and related metabolic complications. There is great interest to use dietary means to manipulate the microbiota to promote health. Currently, the impact of dietary change on the microbiota and the host metabolism is poorly predictable and highly individual. We propose that the responsiveness of the gut microbiota may depend on its composition, and associate with metabolic changes in the host.

**Methodology:**

Our study involved three independent cohorts of obese adults (n = 78) from Belgium, Finland, and Britain, participating in different dietary interventions aiming to improve metabolic health. We used a phylogenetic microarray for comprehensive fecal microbiota analysis at baseline and after the intervention. Blood cholesterol, insulin and inflammation markers were analyzed as indicators of host response. The data were divided into four training set – test set pairs; each intervention acted both as a part of a training set and as an independent test set. We used linear models to predict the responsiveness of the microbiota and the host, and logistic regression to predict responder vs. non-responder status, or increase vs. decrease of the health parameters.

**Principal Findings:**

Our models, based on the abundance of several, mainly Firmicute species at baseline, predicted the responsiveness of the microbiota (AUC  =  0.77–1; predicted vs. observed correlation  =  0.67–0.88). Many of the predictive taxa showed a non-linear relationship with the responsiveness. The microbiota response associated with the change in serum cholesterol levels with an AUC of 0.96, highlighting the involvement of the intestinal microbiota in metabolic health.

**Conclusion:**

This proof-of-principle study introduces the first potential microbial biomarkers for dietary responsiveness in obese individuals with impaired metabolic health, and reveals the potential of microbiota signatures for personalized nutrition.

## Introduction

The gut microbiota is an important contributor to human health, and is emerging as a promising target for therapeutic modulation [1], [2]. Obesity-related diseases offer a prime example where intestinal bacteria have recently been implicated as one etiological factor [3]–[5]; hence modifying the gut microbiota represents a potential strategy for successful treatment [3], [6], [7]. However, it is currently impossible to make practical guidelines as to how the microbiota should be modified. Although recent research has identified compositional and functional properties that characterize the intestinal microbiota in healthy individuals [8], we are lacking a definition for a healthy microbiota, mainly because of the vast inter-individual variation [9]. Furthermore, individuals' responses to dietary interventions are highly variable and poorly predictable – both in terms of host metabolism as well as the gut microbiota – and sometimes even contrary to what was expected from *in vitro* studies [10]–[13]. Hence, the key challenge for the therapeutic modulation of the gut microbiota is to identify individuals who will benefit from a given intervention, with respect to their microbiota composition, and most importantly, with regard to clinical health markers. Personalized nutritional and pharmaceutical therapy, based on information of the individual's gut microbiota, have great prospects in the treatment of obesity and related conditions [10], [14].

We propose that the composition of the gut microbiota may be informative in predicting the responses of the microbiota and of the host to a dietary intervention. Community composition influences the responses of its members to disturbances through ecological and evolutionary interactions [15]; the baseline composition of the gut microbiota is likely to influence the responses of individual bacterial strains, and consequently those of the bacterial community and the host. We test this hypothesis using three independent data sets of obese individuals undergoing different types of dietary interventions, and attempt to predict the responses of both the host and the microbiota.

## Methods

### Participants and dietary interventions

We used three previously published cohorts of Finnish, Belgian and British adults who were obese and/or had metabolic syndrome (n = 78; 71 were obese (BMI over 30 kg/m^2^), and 7 were overweight (BMI 26–29) and had diagnosed metabolic syndrome). All subjects underwent dietary interventions, which altered the quantity and/or quality of ingested carbohydrates and by doing so, aimed for improved metabolic health and reduced risk for type 2 diabetes. The details of the study designs and diets, inclusion and exclusion criteria as well as the analytical procedures can be found in the original publications specified below. We used microbiota and clinical data collected at the beginning and at the end of each trial.

Studies A and B consist of a Finnish 12-week trial with 52 participants (27 females, 25 males, age 40–65, BMI 26–39 kg/m^2^) fulfilling the criteria for metabolic syndrome [16]. The participants were randomized into two intervention groups: one group (n = 28) ate high-fiber rye bread and whole-grain pasta (hereafter referred to as study A), and the other group (n = 24) substituted grains in their habitual diet with low-fiber, refined wheat bread (study B). The samples were frozen in −70°C until DNA extraction with the Repeated Bead Beating method [17].

Study C is a Belgian 12-week trial [18] from which we included the intervention group (n = 13, all females, BMI >30 kg/m2), which received a daily dose of 8g inulin and 8g oligofructose. The fecal samples were stored in −20°C until DNA extraction with the QiAamp Stool DNA Mini Kit (Qiagen, Hilden,Germany). The kit procedure was modified according to Salonen et al. (2010); however, the fecal samples were not beat-beaten, but subjected to mechanical homogenization upon vortex agitation with micro-beads (VWR, Belgium), and the bacterial lysis was improved by heating samples at 95°C for 5 min.

Study D is a British 10-week trial [12] in which the participants (n = 13, all males, age 27–73, BMI 28–51), fulfilling the criteria for metabolic syndrome, consecutively received 3 different diets after a run-in diet for one week. The interventions, each for 3 weeks, included a resistant-starch-enriched diet, a non-starch-polysaccharide-enriched diet, and finally a weight-loss diet, low in carbohydrates and fat, and high in protein. We used the data collected during the run-in diet, and at the end of the weight-loss diet. The DNA was extracted from fresh fecal samples using the FastDNA Spin kit for soil (Qbiogene, Carlsbad, CA).

Total blood cholesterol, HOMA (Homeostastic Model Assessment, indicator of insulin sensitivity), and CRP (C-reactive protein, indicator of systemic inflammation) values, measured before and after the intervention, were available for all studies, except CRP for study D, and were used as markers for host responsiveness to the intervention. Blood sampling and analysis have been described previously for studies A and B [16], study C [18] and study D [19]. Host blood marker values at baseline, and their relative change after intervention are presented in Fig. S1.

As a reference for the microbiota composition and temporal dynamics, we included 15 Finnish healthy normal-weight individuals, not undergoing any intervention, from a previously published study [20]. As obese controls we used the control group from study C (n = 15).

### Intestinal microbiota analysis

All samples were analyzed with the HITChip microarray, which is designed for the analysis of the human gut microbiota, relies on the identification of the V1 and V6 regions on the 16S rRNA gene, and can detect and quantify the relative abundances of over 1000 species-level (L3) phylotypes. These can be summarized into 130 genus-like groups (≥90% sequence similarity in the 16S rRNA gene; referred to as L2) and further to 23 L1 taxa that represent 10 phyla, the Firmicutes being further divided into Clostridium clusters, uncultured Clostridiales and Bacilli [21]. Probe signals summarized to the above-mentioned phylogenetic levels were used as indicators of bacterial abundance. The microbiota data, generated from fecal samples collected before and after the interventions, were extracted using min-max normalization [22] against an in-house data collection of over 5000 microarray experiments [23]. The microarray data are available from the Dryad Digital Repository: http://doi.org/10.5061/dryad.bv4k7. To gain normality, the HITChip hybridization signals were log transformed. The Pearson correlation between the baseline and the post-intervention sample, based on the species-level data, was calculated to define the stability of the microbiota for each individual. The stability was used as an indicator of the microbiota responsiveness to dietary intervention and treated in two ways: as a continuous variable, in which case we attempted to predict the exact stability values, or as a categorical variable, including in the responder group those with Pearson correlation <0.87 (n = 14, 18% of the individuals), and in the non-responder group those with Pearson correlation >0.92 (n = 43, 55%). The cut-off values were based on the distribution of the stability values presented in Fig. S2.

Quantification of methanogenic archaea, not detected by the HITChip, was carried out with quantitative PCR with previously described primers and reaction conditions [17].

### Data normalization

**Figure 1:**
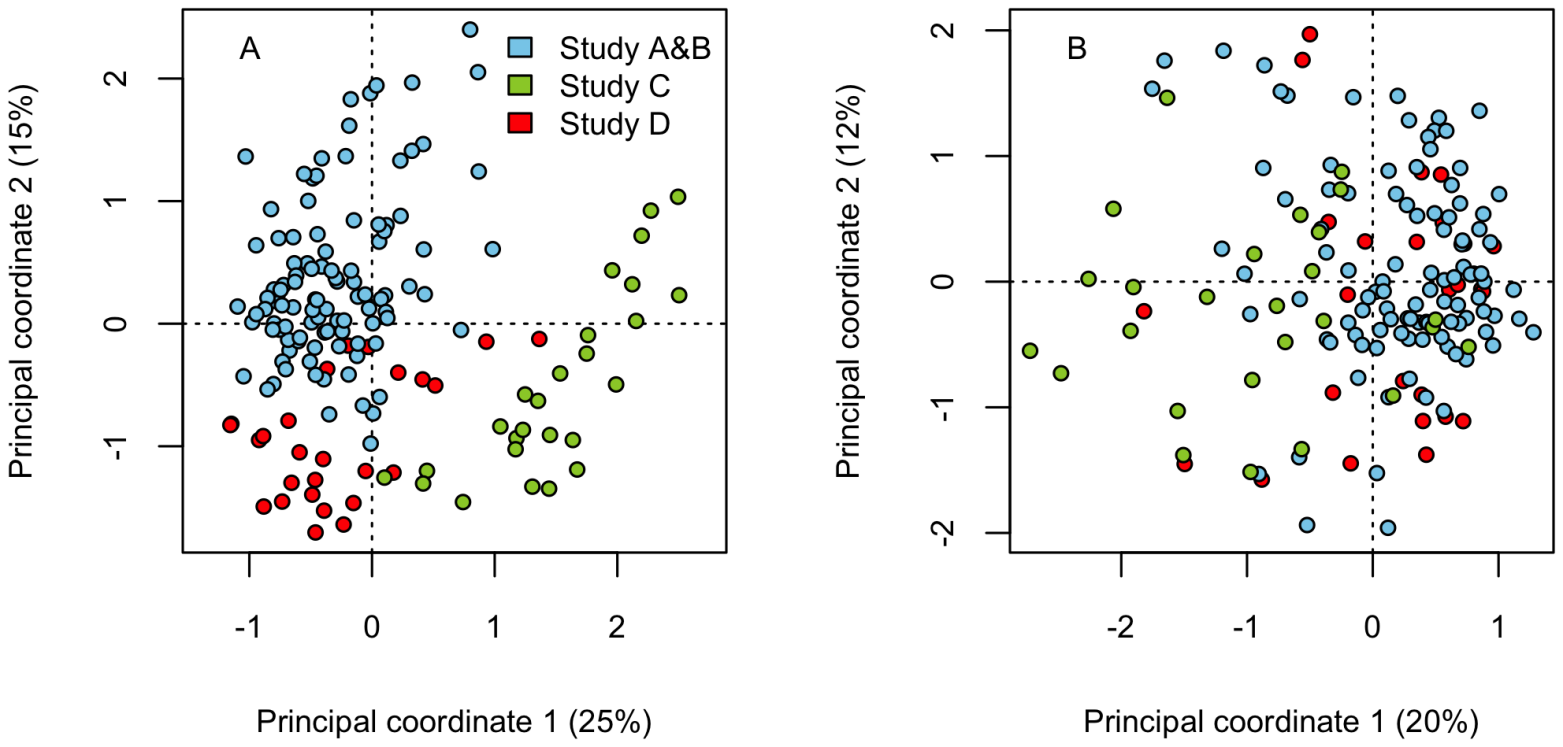
Result of data normalization. Principal co-ordinates plots (with Bray-Curtis distances) show that the microbiota profiles segregate based on the study before (panel A), but not after (panel B) data normalization.

Unsupervised clustering and principal coordinates analysis of the baseline microbiota revealed that the data clustered by study (Fig. 1A). The nature of the observed differences in the microbiota composition between the studies suggested a technical rather than a biological basis: the gram-negative bacteria were elevated, and the gram-positive bacteria reduced in studies C and D compared to studies A and B (Fig. S3). The effect of PCR bias or different analytical procedures can be excluded as all samples were processed similarly for the microarray hybridization. Instead, such differences can arise from the use of differentially efficient DNA extraction methods, as the gram-negative organisms become overrepresented with methods that fail to lyse part of the dominant, more recalcitrant gram-positive bacteria. Such suboptimal performance has been reported for the Qiagen kit [24], even when preceded with short mechanical lysis [17], which was used in study C. Indeed, the overall diversity, measured by the inverse Simpson diversity index, was significantly lower in study C compared to the other studies, suggesting incomplete DNA extraction. Secondly, the relative amount of *Bacteroides* spp. is sensitive to storage conditions; their amount is significantly higher in fresh than frozen samples [24], potentially explaining the higher abundance of Bacteroidetes in samples of study D, which were extracted from fresh samples with mechanical lysis. To eliminate these presumably technical differences that prevented integrated analysis of the cohorts, we normalized the datasets: First, we calculated the total average (log-transformed) signal intensity of each L1 group over all samples (M_T_), and average signal intensities for each L1 group in each study (M_A_, M_B_, M_C_, M_D_). For each L1 group and study, we then calculated the % difference between the total average (M_T_) and the study average as D_A_  =  (M_A_ – M_T_)/M_T_, D_B_  =  (M_B_ – M_T_)/M_T_, D_C_  =  (M_C_ – M_T_)/M_T_, D_D_  =  (M_D_ – M_T_)/M_T_. The normalized L2 and L3 signals were obtained by multiplying the original values with 1-D for the study and respective L1 group. After normalization, the studies no longer separated in PCO (Fig. 1B).

### Model selection and validation

From the total (species- and genus-level) microbiota data, we formed four training-validation data set pairs and performed model selection and validation separately for each data set pair following the same procedure (detailed below). Training set 1 included all studies except Study A, which acted as the validation set; training set 2 included all studies except study B; training set 3 included all but study C; and training set 4 included all but study D. Therefore, we had essentially four training data sets, with four independent validation data sets.

To select and validate the predictive model for microbiota responsiveness, we fitted linear models (separately for each training data set) with the microbiota stability as the response variable and the abundance of each L2 and L3 bacterial group separately as the only explanatory variable, allowing for linear and quadratic relationships. Although linear models assume that the relationship between the predictor and response variable is linear, non-linear relationships can be estimated by including a quadratic term in the model: the relationship between the response variable may be linearly related to the predictor squared, and thus non-linearly (quadratically) related to the predictor. From these models we extracted p-values for the bacterial groups, as indicators of their potential relevance as predictors of microbiota responsiveness. We then built full models separately for each training set, which included all bacterial groups with p-values <0.02, now allowing for interactions between the bacterial groups. These models were then reduced using AIC (Akaike Information Criterion) as the criterion of inclusion/exclusion of variables. Several different penalty values (2–8) were used to arrive at a set of different-sized models. These models were then tested for their ability to predict the independent validation data set by calculating the correlation between the model-predicted and the observed stability values for the validation set. The final best model was chosen as the one, which emerged from all four training data sets, and was adequately able to predict all four validation data sets. The same procedure was conducted with the microbiota responder vs. non-responder categories, using logistic regression. To assess whether the model predicted temporal stability in general, or responsiveness to dietary intervention specifically, we used the model to predict temporal stabilities in the control samples.

Host responsiveness was treated as categorical (>10% increase vs. >10% decrease, excluding the cases with <10% change) and continuous. Model selection and validation for HOMA and CRP responses were conducted as detailed above. In the case of the responses, the different studies were not directly comparable due to different average responses per study. We corrected for this by including the study effect in the cholesterol response models as a fixed term, and performed model selection and validation by dividing the total data set randomly into a training set (75% of the data) and a validation set (25%). We present the combined result of 5 times repeated model validation.

To ensure that the normalization did not confound the analysis, we tested the models with the non-normalized microbiota data. The models performed well for studies A, B, and D. The responses within the study C, which had the most divergent L1 composition, could only be predicted after data normalization (data not shown).

We compared the diversity, richness, and evenness of the microbiota, as well as the presence or absence of methanogenic archaea, and *Bacteroides/Prevotella* ratio with the microbiota and host responsiveness using linear models.

Finally, we compared the baseline abundances of the organisms that were identified as predictive, between our obese cases and healthy controls using analysis of variance.

All analyses were conducted with R [25]. ROC (Receiver Operating Characteristic) curves were calculated with the package pROC [26].

## Results

To study the possibility to differentiate and predict highly individual dietary responses based on the intestinal microbiota, we searched for associations between its baseline composition and the degree of responsiveness of the microbiota, and of the host, to dietary interventions.

### Predicting the microbiota response

**Figure 2:**
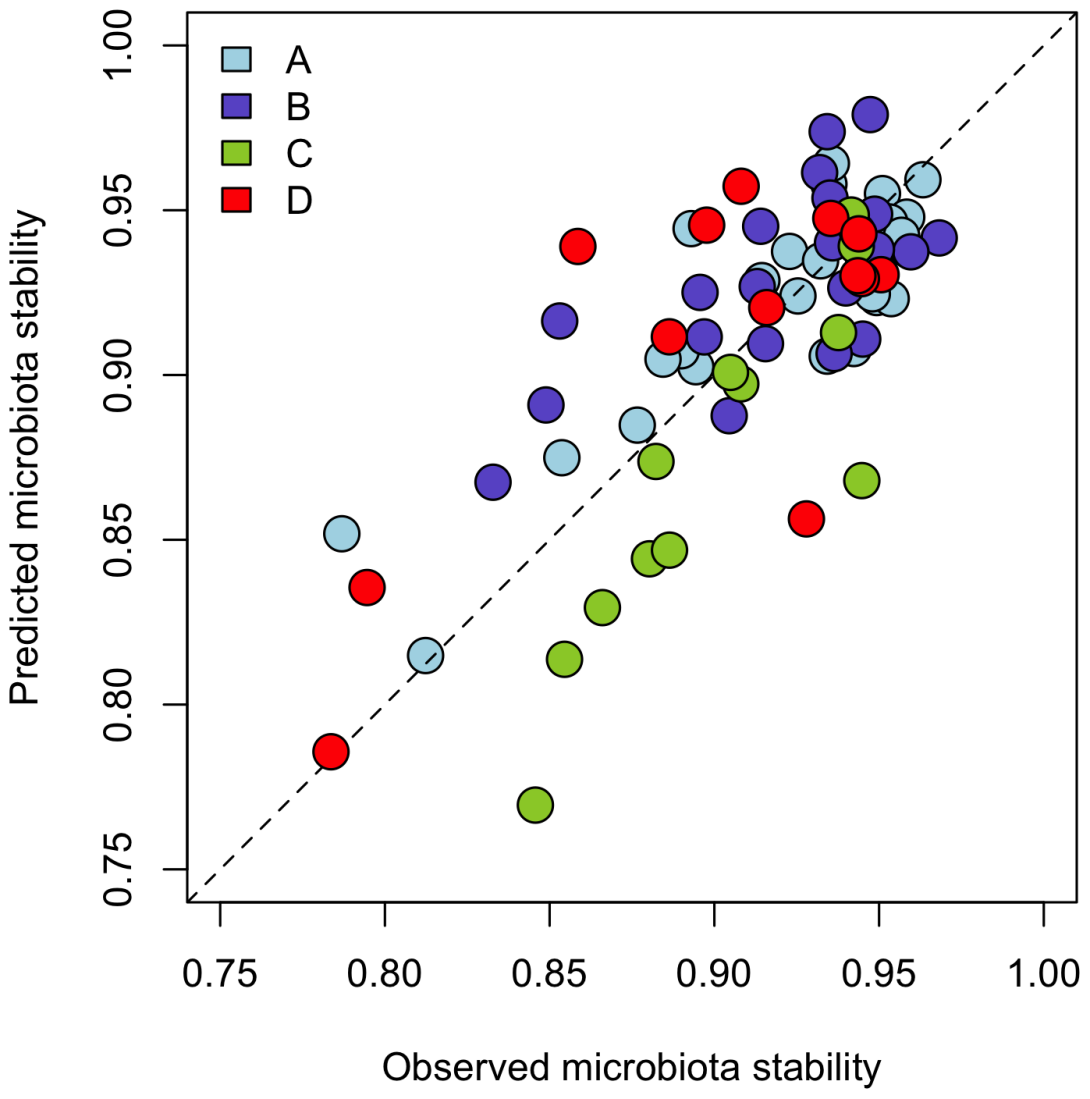
Validation of the microbiota responsiveness model. The model selection and validation were conducted four times, each time leaving out one study (marked with letters A-D). The resulting model was used to predict the stability values in the left-out study. The dashed line represents the ideal situation where observed  =  predicted.

A linear model with the baseline abundances of members of Clostridium clusters IV, IX, and XIVa, and Bacilli (Table 1) was able to predict the overall responsiveness of the gut microbiota to all tested dietary interventions, as demonstrated by the strong correlations between the observed and the model-predicted values of microbiota stability (Fig. 2). The parameter estimates are presented in Table S1.

**Table 1:** Bacterial taxa predictive of microbiota or host responses to dietary interventions.

Predictive taxon	NCBI Accession#	Genus-like group	Firmicute group or phylum	Predicts the response of	Characteristics	Average abundance	Obese vs. Control
*Dialister*	AY162469	*Dialister*	CC IX	Microbiota	Predictive of host IL-6 response to dietary intervention (1)	0.90%	NS
*Lactococcus* sp 451	AY762109	*Lactococcus*	Bacilli	Microbiota	Abundant in fermented dairy products; biological response modifier (2)	<0.01%	Decreased
*Oscillospira guillermondii* et rel.		*O. guillermondii* et rel.	CC IV	Microbiota	In MZ-twins, associated with low BMI difference (3)	9.54%	NS
Uncultured bacterium K305	AY916194	*Clostridium colinum* et rel.	CC XIVa	Microbiota		0.01%	Decreased
Uncultured bacterium K375	AY916197	*Clostridium symbiosum* et rel.	CC XIVa	Microbiota		0.18%	NS
Uncultured *Selenomonadaceae*	AY684401	Uncultured *Selenomonadaceae*	CC IX	Microbiota	Common gut inhabitant, abundant in animals on a high-starch diet, utilizes saccharides and lactate (4)	0.02%	NS
*Clostridium felsineum*	AF270502	*C. felsineum* et rel.	CC XI	Microbiota& Cholesterol	Hydrolytic, produces butyrate (8)	<0.01%	Decreased
*Clostridium sphenoides*	AB075772	*C. sphenoides* et rel.	CC XIVa	Microbiota& cholesterol	Common gut inhabitant (5)	0.01%	Decreased
*Eubacterium ruminantium*	AB008552	*Coprococcus eutactus* et rel.	CC XIVa	Microbiota& cholesterol	Common gut inhabitant, hydrolytic, produces butyrate, lactate, formate (4)	0.01%	Decreased
Bacterium adhufec335	AF132262	*Eubacterium ventriosum* et rel.	CC XIVa	HOMA		0.01%	NS
*Eubacterium biforme*	M59230	*E. biforme* et rel.	CC XVI	HOMA	Common gut inhabitant, produces butyrate, H2, lactate (4).	0.02%	NS
*Eubacterium contortum*	L34615	*Ruminococcus gnavus* et rel.	CC XIVa	HOMA	Common gut inhabitant, found in human infections, produces H2, ethanol, formate (4)	0.01%	Decreased
*Lactobacillus buchneri*	M58811	*Lactobacillus plantarum* et rel.	Bacilli	HOMA	Cholesterol-lowering properties (6), obligately heterofermentatitve (4)	0.01%	Decreased
*Sutterella wadsworthia*	L37786	*S. wadsworthia* et rel.	Proteobacteria	HOMA	Abundant in small intestinal mucosa of healthy and diseased individuals (7,9)	0.04%	NS
Uncultured bacterium M615	AY916164	*Bulleidia moorei* et rel.	CC XVI	HOMA		<0.01%	NS
Bacterium adhufec250	AF132253	*Bryantella formatexigens* et rel.	CC XIVa	CRP		0.09%	NS
*Clostridium ramosum* et rel.	X73440	*C. ramosum* et rel.	CC XVIII	CRP	Common gut inhabitant (5)	0.06%	NS
*Lachnobacterium bovis* et rel.		*L. bovis* et rel.	CC XIVa	CRP Produces lactate and bacteriocins (10)		0.71%	Decreased
Diversity of CC XI				CRP			NS
Uncultured bacterium cadhufec32c10	AF530372	*Papillibacter cinnamivorans* et rel.	CC IV	CRP		0.01%	Decreased
Uncultured bacterium D416	AY916368	*Bryantella formatexigens* et rel.	CC XIVa	CRP		0.29%	NS
Uncultured *Leuconostoc* sp. LabS38	AF335916	*Weissella*	Bacilli	CRP		0.01%	NS

Taxa which were identified as predictive of host or microbiota responses to dietary interventions.

CC =  Clostridium Cluster. 1)[45]; 2)[46]; 3)[47]; 4)[48]; 5)[49]; 6)[50]; 7)[51]; 8)[37]; 9)[52]; 10)[53].

When treating the responsiveness as a categorical variable, and including only the clear responders (stability <0.87) and clear non-responders (stability > 0.92; Fig. S2), the model with the baseline abundances of *Eubacterium ruminantium* and *Clostridium felsineum* was able to predict with great accuracy all independent data sets (Fig. S4): AUC (Area Under the Curve) for study A  =  98.15% (95% confidence interval: 93.02%–100%); study B  =  77.78% (47.92%–100%); study C  =  100% (100%–100%); study D  =  94.44% (79.05%–100%). The non-responders were characterized by average abundances of both species, while the responders had either very low or very high baseline abundances of *E. ruminantium* plus *C. felsineum* (Fig. S5).

Neither the linear nor the logistic model was able to predict the microbiota stability of the control cases (study C, obese controls, data not shown); the model specifically predicted responses to dietary interventions.

Finally, we were interested in identifying the bacterial groups, which could predict the change in bifidobacterial abundance, as many of the diets strongly affected bifidobacteria in some, but not all individuals. The direction and magnitude of change in bifidobacteria was correlated only with their own baseline abundance (Pearson correlation  =  −0.40, p<0.0001; Fig. S6), indicating that intestinal bifidobacterial populations are strongly regulated by negative density dependence.

### Predicting the host response

**Figure 3:**
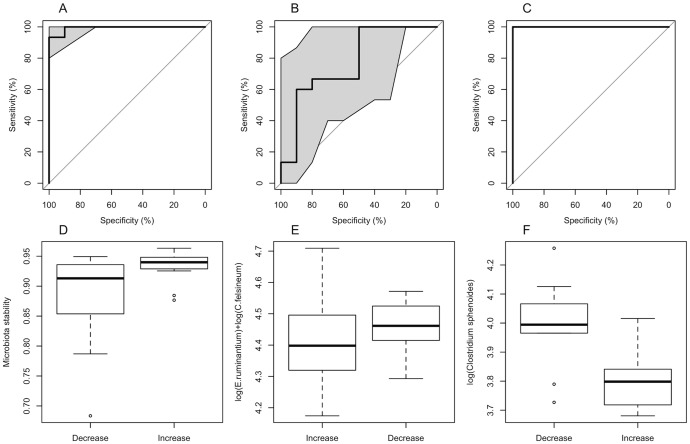
Predicting cholesterol responses to dietary intervetions. Panels A, B, C: Three cholesterol response models: cholesterol response predicted by the microbiota stability (panel A), by the baseline abundance of *E. ruminantium* and *C. felsineum* (B), and by the baseline abundance of *C. sphenoides* (C). The data were divided randomly into a training set (75% of the data) and test set (the remaining 25%), and the ROC curves represent the ability of the models, fitted to the training data, to predict the cholesterol response (increase vs. decrease) in the test data. The ROC curve shows the true positive rate ( = sensitivity) against the false positive rate ( = 1-specificity) for the different possible cut points of a diagnostic test. The perfect diagnostic test would have a sensitivity  =  1 and specificity  =  1, and therefore the area under the curve (AUC) would be 1. A random guess would have a ROC curve following the diagonal; curves above the diagonal indicate that the classifier works better than a random guess. Shaded areas represent 95% confidence intervals for the ROC curve. Panels D, E, F: Comparison of cholesterol response groups (increase vs. decrease), with respect to microbiota stability (D), *E. ruminantium* and *C. felsineum* abundance (E), and *C. sphenoides* abundance (F).

The cholesterol, HOMA, and CRP responses varied widely (Fig. S1), but were not interrelated. The cholesterol response was related to the overall microbiota responsiveness, as the individuals with a responsive microbiota all showed either a decrease (39%) or no marked change (62%) in cholesterol levels, while only 21% of the individuals with a non-responsive microbiota showed a decrease in cholesterol levels, and 23% showed an increase. The stability of the microbiota predicted the cholesterol response in the randomly selected validation data set (with different intercepts for different studies) with an AUC of 96% (95% CI: 89.33%–100%, Fig. 3A, D). Moreover, the same species, which predicted the microbiota response (*E. ruminantium* and *C. felsineum*), predicted the cholesterol response with an AUC of 82.67% (65.17%–100%, Fig. 3B, E). Finally, a model with only the abundance of the species *Clostridium sphenoides* and different intercepts for the different studies, predicted the cholesterol response with an AUC of 100% (100%–100%). The abundance of *C. sphenoides* was significantly (p<0.05) lower in the individuals with an increase in cholesterol levels, as compared to those with a decrease (Fig. 3C, F).

**Figure 4:**
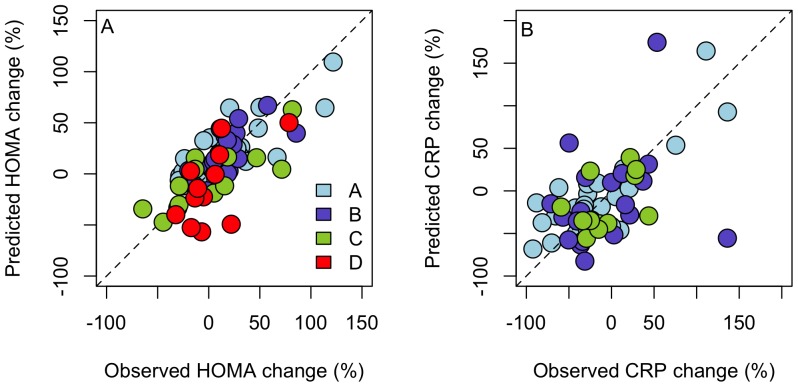
Validation of the HOMA (panel A) and CRP (panel B) response models. In each case, one study was left out, while data from the other studies were fitted to the model, which was then used to predict the HOMA and CRP response for the independent data set (A–D). The dashed line represents the ideal situation where observed  =  predicted.

The HOMA response was not linked to the microbiota response, but was predicted by a model including the baseline abundances of members of Clostridium clusters XVI, and XVIa, Bacilli, and Proteobacteria (Table 1, Table S1). The correlations between the predicted and observed HOMA responses were between 0.56 and 0.79 in the different validation data sets (Fig. 4A).

The CRP response was independent of the microbiota response, but was predicted by a model including the baseline abundances members of Clostridium clusters VI, XI, XIVa, and XVIII (Table 1, Table S1). The correlations between the predicted and observed CRP responses were between 0.46 and 0.80 in the different validation data sets (Fig. 4B).

The diversity, richness, or evenness of the microbiota, or the carriage of methanogenic archaea was not associated with responsiveness (data not shown), nor was the *Bacteroides/Prevotella* ratio (Pearson correlation  =  0.06, p = 0.52; Fig. S7).

To confirm that the results were not platform-specific, we included pyrosequencing data in the analysis. The data were derived from fecal samples collected from 28 healthy adults (mean BMI 25) before and after a four-week intervention on brown rice and whole grain barley [27]. Most of the predictive bacteria identified with the HITChip as predictors were not detected in this data set, probably due to their low abundance (Table 1), so we were unable to test the models with the sequencing data. However, for those bacteria, which were detected, the relationship with the microbiota responsiveness were comparable to that found in the HITChip data (Fig. S8).

## Discussion

### The prognostic value of the gut microbiota

This is the first study to explicitly address the individual-specific responses of the human microbiota to interventions, a long-know phenomenon, which has, to date, been treated largely as random noise. Our work revealed that rather than being random, the response of the gut microbiota to dietary interventions can be predicted with high accuracy based on the initial microbiota composition. Previously, the gut microbiota composition has been used to successfully differentiate individuals with type 2 diabetes [28], [29] and IBD [30] from healthy controls, but this is the first study to demonstrate the prognostic value of the gut microbiota.

Obesity is a multifactorial state, where host genes, life style and, as recently identified, the gut microbiota [4], [5] interact in a complex and largely unknown way. Predicting how an individual will respond to a dietary intervention is a major challenge with the potential to revolutionize the management of obesity and associated pathologies. Previously, adipose gene expression profiles have been used to predict weight loss response with 80% accuracy [31]. We have, for the first time, provided evidence that intestinal bacteria, our microbial metabolic organ [4], can be used to predict the host's metabolic response to a dietary intervention. These results were found to apply to different types of dietary interventions, ranging from a simple addition of a prebiotic compound (study C), to a change in the type of grains in the diet (studies A and B), to a dietary change entailing profoundly altered macronutrient composition (study D). It remains to be studied whether the gut microbiota composition can be used to predict the response to other types of dietary changes, e.g. in fat content.

### Microbiota and host responses are interconnected

Our results indicate that some obese individuals gain health benefits from a very simple and easily managed dietary change, while others show no or even adverse responses, and may require more profound treatment approaches. In this cohort, the cholesterol responses were associated with the responsiveness of the gut microbiota: a change in the gut microbiota appeared to be necessary for the cholesterol values to lower. Similarly, Faith et al. (2013) reported, based on sequencing data of healthy US adults, that the change in BMI was associated with changing gut microbiota [32]. Overall, our results confirm the previously found link between the gut microbiota and host lipid metabolism [33], [34], and suggest that the successful improvement of lipid metabolism is associated, and possibly dependent on, a change in the gut microbiota composition.

The responsiveness of the microbiota appears to be a separate phenomenon from the temporal dynamics in the absence of intervention, as our models were unable to predict the temporal stability of the microbiota in control individuals. This suggests that these two traits are determined by different factors. Responsiveness to a dietary change may, for example, reflect the primary response of nutritionally specialized microbes, or indirect effects due to cross feeding and/or competition. Temporal dynamics in the absence of any specific stimulator or disturbance, in turn, may reflect e.g. oscillatory dynamics due to density-dependent feedback (see 4.4) or other reasons.

### Predictive organisms may be bioindicators

Most strikingly, the cholesterol response could be predicted from the abundance of a single species, *Clostridium sphenoides,* measured from the fecal sample before the dietary intervention. A decrease in cholesterol levels was observed mostly among the individuals with high *C. sphenoides* abundance. Furthermore, the abundance of *C. sphenoides* was in general decreased in our obese study subjects as compared to healthy controls (Table 1). Obese individuals with a “healthy” abundance of *C. sphenoides* thus appear to benefit even from simple dietary interventions in terms of lipid metabolism, while those with abnormally low abundance do not. The abundance of *C. sphenoides* was not associated with the absolute levels of cholesterol (data not shown), and therefore may not be directly associated with cholesterol metabolism, but may rather be an indicator of a gut ecosystem which, upon improved diet, can contribute positively to host lipid metabolism.

Very little is known about the two organisms, which predicted the responsiveness of the microbiota (*C. felsineum* and *E. ruminantium*). *E. ruminantium* belongs to the family *Lachnospiraceae*, has originally been isolated from bovine rumen, but is also part of the human intestinal microbiota [35]. It is xylanolytic and produces mainly formic acid, but also butyrate [36]. *C. felsineum* (family *Clostridiaceae)* is a pectinolytic butyrate-producer [37]. Hence, both bacteria occupy the most common niche in the gut, degradation and fermentation of indigestible carbohydrates.

The predictive bacteria identified in this study were present at a very low abundance. Only the relative abundance of *Oscillospira guillermondii-*group, which itself was not predictive but modulated the effects of the predictive organisms (Table S1), was above 1% (Table 1). While high analytical depth is required to detect such minorities, their functional relevance should not be overlooked. As an example, the acetogens, methanogenic archaea, and sulfate-reducing bacteria, which dispose the colonic hydrogen gas generated during fermentation, are low in abundance, but critical for the functioning of the gut ecosystem [38]. It is very likely that the organisms we found are not *per se* causative of the responsiveness (of the host or the microbiota), but may rather be indicator species, particularly sensitive to the environment and therefore informative of important structural or functional differences between ecosystems, which lead to the differential responses. We acknowledge that the accurate identification of species-level phylotypes with the microarray cannot be ascertained, and hence the true identities of the implicated organisms need to be validated in further studies.

Clostridial species dominate the list of predictive organisms (Table 2). Bacteroidetes were notably non-predictive, as was the *Bacteroides/Prevotella* ratio. This is somewhat surprising as both of these genera are, in parallel to above-mentioned *Clostridiales*, active degraders of dietary polysaccharides that were essential components of all intervention diets. The finding is interesting also in the light of the discussion about enterotypes, which have been defined largely by the abundance of the genera *Bacteroides* and *Prevotella* [39]. Our findings suggest that the major determinants of the inter-individual differences of the gut microbiota may not be the most relevant for predictive purposes. Microbiota richness has been positively associated with the microbiota responsiveness to weight loss diets in obese individuals [40], but in our study, species richness, or diversity, was not associated with the responsiveness. However, most of the diets in our study were not weight-loss diets, which may explain the difference.

**Table 2:** Predictive organisms are mostly clostridia.

L1	Phylotypes per L1 group (% of total)	Predictive phylotypes (% of all predictors)	Odds ratio	p
Bacilli	74 (8%)	3 (14%)	1.72	NS
Clostridium cluster IV	175 (19%)	2 (9%)	0.47	NS
Clostridium cluster IX	27 (3%)	2 (9%)	3.25	NS
Clostridium cluster XI	24 (3%)	2 (9%)	3.69	NS
Clostridium cluster XVIII	5 (1%)	1 (5%)	10.11	NS
Clostridium cluster XIVa	221 (24%)	9 (41%)	1.72	NS
Clostridium cluster XVI	10 (1%)	2 (9%)	10.11	<0.05
CC IX, XI, XVIII, XVIa, XVI	287 (31%)	16 (73%)	2.41	<0.05
Proteobacteria	98 (11%)	1 (5%)	0.42	NS

Numbers of predictive organisms per each implicated high-level phylogenetic group (L1, see section 2.2. for explanation), compared to the total number of species in the L1 group. Odds ratio >1 indicates that the group contains more predictive phylotypes than expected based on the total number of phylotypes in the group. Statistical significance of the odds ratio was estimated with the Fisher's test.

### The importance of non-linear relationships and density dependence

Many of the predictive taxa showed non-linear associations with the host and microbiota responsiveness, which would have been missed, had we allowed only linear associations. Non-linear relationships abound in nature. For example, species responses to environmental gradients are very often unimodal, rather than linear [41]: there is a certain preferred level, below and above which the species does poorly. Instead of the low vs. high abundance of a given bacterium, we found that the important distinction was often between individuals with average vs. extreme, either low or high, abundances. It is possible that the extreme abundances of the identified predictor species indicate a shift in ecosystem function, and the *magnitude* of the shift, rather than the *direction*, is of prognostic relevance. A disturbance may reduce the abundances of some species, allowing others to overgrow. The direction of the shift in competitive balance may be relatively random between individuals, depending on subtle differences in the ecosystem structure, and hence, may be less important than the magnitude.

Moreover, we present evidence of negative density dependence regulating the bacterial populations in the human intestine: The lower the baseline abundance of *Bifidobacterium* spp., the more they increased during the interventions, and vice versa (Fig. S6). This is a long-known phenomenon observed in prebiotic interventions aiming for specific increase of bifidobacteria [42]–[44]. These results indicate that ecological interactions within the microbiota, such as intra-specific competition or phage density, act in parallel to the intervention effects, or even override them. Yet, the importance of baseline abundances have so far been ignored in the community-wide microbiota analyses following dietary interventions. Negative density dependence was evident in all bacterial groups, not only in bifidobacteria (data not shown), which explain more generally why certain intestinal bacteria respond to dietary changes in some, but not all individuals, as noted in numerous studies (e.g. [16], [18], [45]). Hence, when assessing the effect of an intervention on a given bacterial group, we recommend including the baseline abundance in the analysis to control for the impact of density dependence.

### Data normalization

From the methodological perspective, our study is the first to demonstrate how the knowledge of sample processing effects can be utilized retrospectively, enabling meta-analysis or comparison of samples that have been treated differently in the pre-analytical phase. In this study, all samples were analyzed with the same microarray platform with identical primers and workflow. Therefore, the observed differences in the relative share of gram-negative and gram-positive bacteria are likely to originate from differences in DNA extraction and storage procedures. As true biological differences cannot be excluded, the validity of our normalization approach should be confirmed experimentally e.g. in the context of the International Human Microbiome Standards-project (http://www.microbiome-standards.org/). Especially in the absence of standardized procedures, validated data normalization represents an attractive strategy to facilitate efficient and reliable use of the accumulating wealth of human microbiome data sets.

### Concluding remarks

In summary, we present evidence that it is possible to identify obese individuals who will benefit most from a simple dietary intervention based on the gut microbiota composition before the intervention. Clostridial species, in particular, were indicative of the amenability of the gut microbiota to dietary modification, which in turn was associated with the host's lipid metabolism. This concept opens potential new avenues for understanding and treating many disorders. Moreover, it is highly likely that the same approach can be used to identify microbial signatures, which potentially predict the response to other perturbations, such as antibiotics. It should be noted that our analysis represents a proof-of-principle study. Hence, these findings do not yet support any clinical application, but are the first step towards it.

## Supporting Information

Figure S1 **Histograms of the cholesterol (A), CRP (B), and HOMA (C) values of all participants.**
(TIFF)

Figure S2 **Distribution of the microbiota stability values, measured for each individual by calculating the Pearson correlation for the microbiota profiles collected before and after the dietary intervention.** Cut-offs used to classify responders (stability <0.87) and non-responders (stability >0.92) are marked with vertical lines.
(TIFF)

Figure S3 **Relative abundances of the dominant phyla in the different studies (A–D) before data normalization.**
(TIFF)

Figure S4 **Model validation for the classification of individuals into microbiota responders and non-responders based on baseline microbiota.** The model was fitted to four training data sets and used to predict four validation data sets A–D, shown in the different panels. The ROC curves present the model performance in each training-validation data set pair (shaded areas represent the 95% confidence intervals).
(TIFF)

Figure S5 **The summed abundance of two clostridial species differentiates microbiota-responders from non-responders; responders have either very low or very high abundances, while non-responders have average abundances of these organisms.**
(TIFF)

Figure S6 **The change in the abundance of bifidobacteria is associated with the baseline abundance of bifidobacteria.**
(TIFF)

Figure S7 **The baseline** ***Bacteroides/Prevotella*** **ratio is not associated with the microbiota response to dietary interventions.**
(TIFF)

Figure S8 **Relationship between the abundance of uncultured bacterium K375 and** ***Dialister*** **spp., with microbiota stability, measured with the HITChip in European studies and by 454 sequencing in an American study.** The average abundances and stabilities were not comparable between platforms and were therefore scaled to mean  =  0, and sd  =  1.
(TIFF)

Table S1 **Parameter estimates of the models for microbiota and host responses.** The larger the estimate, the stronger the effect; negative values indicate a negative relationship and *vice versa*. The intercept is the estimated value of the response variable when all predictors are 0.
(DOCX)

## References

[1] Bäckhed F (2012) Host responses to the human microbiome. Nutr Rev 70: S14–S17. 22861802 10.1111/j.1753-4887.2012.00496.x

[2] Holmes E, Kinross J, Gibson GR, Burcelin R, Jia W, et al (2012) Therapeutic modulation of microbiota-host metabolic interactions. Science Translational Medicine 4: 137rv6. 10.1126/scitranslmed.3004244 22674556

[3] Million M, Lagier J, Yahav D, Paul M (2013) Gut bacterial microbiota and obesity. Clinical Microbiology and Infection 19: 305–313. 23452229 10.1111/1469-0691.12172

[4] Everard A, Cani PD (2013) Diabetes, obesity and gut microbiota. Best Practice & Research Clinical Gastroenterology 27: 73–83. 23768554 10.1016/j.bpg.2013.03.007

[5] Flint HJ (2011) Obesity and the gut microbiota. J Clin Gastroenterol 45: S128–S132. 21992951 10.1097/MCG.0b013e31821f44c4

[6] Kootte RS, Vrieze A, Holleman F, Dallinga-Thie GM, Zoetendal EG, et al (2012) The therapeutic potential of manipulating gut microbiota in obesity and type 2 diabetes mellitus. Diabetes Obesity & Metabolism 14: 112–120. 10.1111/j.1463-1326.2011.01483.x 21812894

[7] Delzenne NM, Neyrinck AM, Baeckhed F, Cani PD (2011) Targeting gut microbiota in obesity: Effects of prebiotics and probiotics. Nature Reviews Endocrinology 7: 639–646. 10.1038/nrendo.2011.126 21826100

[8] Walker AW, Lawley TD (2012) Therapeutic modulation of intestinal dysbiosis. Pharmacological Research 69: 75–86. 23017673 10.1016/j.phrs.2012.09.008

[9] Lozupone CA, Stombaugh JI, Gordon JI, Jansson JK, Knight R (2012) Diversity, stability and resilience of the human gut microbiota. Nature 489: 220–230. 22972295 10.1038/nature11550 PMC3577372

[10] Lampe JW, Navarro SL, Hullar MAJ, Shojaie A (2013) Inter-individual differences in response to dietary intervention: Integrating omics platforms towards personalised dietary recommendations. Proc Nutr Soc 72: 207–218. 23388096 10.1017/S0029665113000025 PMC3694579

[11] Louis P (2012) Dietary modulation of the human gut microbiota. Agro Food Industry Hi-Tech 23: 26–28.

[12] Walker AW, Ince J, Duncan SH, Webster LM, Holtrop G, et al (2011) Dominant and diet-responsive groups of bacteria within the human colonic microbiota. Isme Journal 5: 220–230. 20686513 10.1038/ismej.2010.118 PMC3105703

[13] McOrist AL, Miller RB, Bird AR, Keogh JB, Noakes M, et al (2011) Fecal butyrate levels vary widely among individuals but are usually increased by a diet high in resistant starch. J Nutr 141: 883–889. 21430242 10.3945/jn.110.128504

[14] Haiser HJ, Turnbaugh PJ (2012) Is it time for a metagenomic basis of therapeutics? Science 336: 1253–1255. 22674325 10.1126/science.1224396

[15] Harmon JP, Moran NA, Ives AR (2009) Species response to environmental change: Impacts of food web interactions and evolution. Science 323: 1347–1350. 19265021 10.1126/science.1167396

[16] Lappi J, Salojärvi J, Kolehmainen M, Mykkänen H, Poutanen K, et al (2013) Intake of whole-grain and fiber-rich rye bread versus refined wheat bread does not differentiate intestinal microbiota composition in Finnish adults with metabolic syndrome. J Nutr 143: 648–655. 23514765 10.3945/jn.112.172668

[17] Salonen A, Nikkila J, Jalanka-Tuovinen J, Immonen O, Rajilic-Stojanovic M, et al (2010) Comparative analysis of fecal DNA extraction methods with phylogenetic microarray: Effective recovery of bacterial and archaeal DNA using mechanical cell lysis. J Microbiol Methods 81: 127–134. 20171997 10.1016/j.mimet.2010.02.007

[18] Dewulf EM, Cani PD, Claus SP, Fuentes S, Puylaert PG, et al (2013) Insight into the prebiotic concept: Lessons from an exploratory, double blind intervention study with inulin-type fructans in obese women. Gut 62: 1112–1121. 23135760 10.1136/gutjnl-2012-303304 PMC3711491

[19] Lobley GE, Holtrop G, Bremner DM, Calder AG, Milne E, et al (2013) Impact of short term consumption of diets high in either non-starch polysaccharides or resistant starch in comparison with moderate weight loss on indices of insulin sensitivity in subjects with metabolic syndrome. Nutrients 5: 2144–2172. 23752495 10.3390/nu5062144 PMC3725498

[20] Jalanka-Tuovinen J, Salonen A, Nikkilä J, Immonen O, Kekkonen RA, et al (2011) Intestinal microbiota in healthy adults: Temporal analysis reveals individual and common core and relation to intestinal symptoms Plos One. 6: e23035. 10.1371/journal.pone.0023035 PMC3145776 21829582

[21] Rajilić-Stojanović M, Heilig HGHJ, Molenaar D, Kajander K, Surakka A, et al (2009) Development and application of the human intestinal tract chip, a phylogenetic microarray: Analysis of universally conserved phylotypes in the abundant microbiota of young and elderly adults. Environ Microbiol 11: 1736–1751. 19508560 10.1111/j.1462-2920.2009.01900.x PMC2784037

[22] Bolstad BM, Irizarry RA, Åstrand M, Speed TP (2003) A comparison of normalization methods for high density oligonucleotide array data based on variance and bias. Bioinformatics 19: 185–193. 12538238 10.1093/bioinformatics/19.2.185

[23] Nikkilä J, de Vos WM (2010) Advanced approaches to characterize the human intestinal microbiota by computational meta-analysis. J Clin Gastroenterol 44: S2–S5. 20616744 10.1097/MCG.0b013e3181e5018f

[24] Maukonen J, Simoes C, Saarela M (2012) The currently used commercial DNA-extraction methods give different results of clostridial and actinobacterial populations derived from human fecal samples. FEMS Microbiol Ecol 79: 697–708. 22098067 10.1111/j.1574-6941.2011.01257.x

[25] R Development Core Team (2011) R: A language and environment for statistical computing Vienna, Austria: R Foundation for Statistical Computing.

[26] Robin X, Turck N, Hainard A, Tiberti N, Lisacek F, et al (2011) pROC: An open-source package for R and S plus to analyze and compare ROC curves. BMC Bioinformatics 12: 77. 21414208 10.1186/1471-2105-12-77 PMC3068975

[27] Martínez I, Lattimer JM, Hubach KL, Case JA, Yang J, et al (2012) Gut microbiome composition is linked to whole grain-induced immunological improvements. The ISME Journal 7: 269–280. 23038174 10.1038/ismej.2012.104 PMC3554403

[28] Karlsson FH, Tremaroli V, Nookaew I, Bergström G, Behre CJ, et al (2013) Gut metagenome in european women with normal, impaired and diabetic glucose control. Nature 498: 99–103. 23719380 10.1038/nature12198

[29] Qin J, Li Y, Cai Z, Li S, Zhu J, et al (2012) A metagenome-wide association study of gut microbiota in type 2 diabetes. Nature 490: 55–60. 23023125 10.1038/nature11450

[30] Papa E, Docktor M, Smillie C, Weber S, Preheim SP, et al (2012) Non-invasive mapping of the gastrointestinal microbiota identifies children with inflammatory bowel disease. Plos One 7: e39242. 22768065 10.1371/journal.pone.0039242 PMC3387146

[31] Mutch DM, Temanni MR, Henegar C, Combes F, Pelloux V, et al (2007) Adipose gene expression prior to weight loss can differentiate and weakly predict dietary responders. Plos One 2: e1344. 18094752 10.1371/journal.pone.0001344 PMC2147074

[32] Faith JJ, Guruge JL, Charbonneau M, Subramanian S, Seedorf H, et al (2013) The long-term stability of the human gut microbiota. Science 341: 1237439. 23828941 10.1126/science.1237439 PMC3791589

[33] Lahti L, Salonen A, Kekkonen RA, Salojärvi J, Jalanka-Tuovinen J, et al (2013) Associations between the human intestinal microbiota, lactobacillus rhamnosus GG and serum lipids indicated by integrated analysis of high-throughput profiling data. PeerJ 1: e32. 23638368 10.7717/peerj.32 PMC3628737

[34] Velagapudi VR, Hezaveh R, Reigstad CS, Gopalacharyulu P, Yetukuri L, et al (2010) The gut microbiota modulates host energy and lipid metabolism in mice. J Lipid Res 51: 1101–1112. 20040631 10.1194/jlr.M002774 PMC2853437

[35] Moore W, Holdeman LV (1974) Human fecal flora: The normal flora of 20 japanese-hawaiians. Appl Microbiol 27: 961–979. 4598229 10.1128/am.27.5.961-979.1974 PMC380185

[36] Louis P, Duncan SH, McCrae SI, Millar J, Jackson MS, et al (2004) Restricted distribution of the butyrate kinase pathway among butyrate-producing bacteria from the human colon. J Bacteriol 186: 2099–2106. 15028695 10.1128/JB.186.7.2099-2106.2004 PMC374397

[37] Rainey FA, Hollen BJ, Small A (2009) Genus I. clostridium. In: De Vos P, Garrity GM, Jones D, Krieg NR, Ludwig W, et al., editors. Bergey's manual of systematic bacteriology. Springer. pp. 774–781.

[38] Nakamura N, Lin HC, McSweeney CS, Mackie RI, Gaskins HR (2010) Mechanisms of microbial hydrogen disposal in the human colon and implications for health and disease. Food Science and Technology 1: 363–395. 10.1146/annurev.food.102308.124101 22129341

[39] Arumugam M, Raes J, Pelletier E, Le Paslier D, Yamada T, et al (2011) Enterotypes of the human gut microbiome. Nature 473: 174–180. 21508958 10.1038/nature09944 PMC3728647

[40] Cotillard A, Kennedy SP, Kong LC, Prifti E, Pons N, et al (2013) Dietary intervention impact on gut microbial gene richness. Nature 500: 585–858. 23985875 10.1038/nature12480

[41] Rydgren K, Økland RH, Økland T (2003) Species response curves along environmental gradients. A case study from SE norwegian swamp forests. Journal of Vegetation Science 14: 869–880.

[42] Davis L, Martínez I, Walter J, Hutkins R (2010) A dose dependent impact of prebiotic galactooligosaccharides on the intestinal microbiota of healthy adults. Int J Food Microbiol 144: 285–292. 21059476 10.1016/j.ijfoodmicro.2010.10.007

[43] Kolida S, Meyer D, Gibson G (2007) A double-blind placebo-controlled study to establish the bifidogenic dose of inulin in healthy humans. Eur J Clin Nutr 61: 1189–1195. 17268410 10.1038/sj.ejcn.1602636

[44] Bouhnik Y, Raskine L, Simoneau G, Vicaut E, Neut C, et al (2004) The capacity of nondigestible carbohydrates to stimulate fecal bifidobacteria in healthy humans: A double-blind, randomized, placebo-controlled, parallel-group, dose-response relation study. Am J Clin Nutr 80: 1658–1664. 15585783 10.1093/ajcn/80.6.1658

[45] Martinez I, Kim J, Duffy PR, Schlegel VL, Walter J (2010) Resistant starches types 2 and 4 have differential effects on the composition of the fecal microbiota in human subjects. PLoS One 5: e15046. 21151493 10.1371/journal.pone.0015046 PMC2993935

[46] Maeda M, Ueda H, Yamazaki M, Ohtsuka M, Doi U (1998) Biological response modifier activity of lactococcus lactis 332. Yakugaku Zasshi-Journal of the Pharmaceutical Society of Japan 118: 150–157. 9564792 10.1248/yakushi1947.118.4_150

[47] Tims S, Derom C, Jonkers DM, Vlietinck R, Saris WH, et al (2013) Microbiota conservation and BMI signatures in adult monozygotic twins. The ISME Journal 7: 707–717. 23190729 10.1038/ismej.2012.146 PMC3603393

[48] Dworkin M, Falcom S, Rosenberg E, Schleifer K, Stackebrandt E, editors (2006) The prokaryotes A handbook on the biology of bacteria. New York: Springer.

[49] Moosavian M, Hayati K (2008) A survey of clostridia in the patients with acute diarrhea compared with the control group. Pakistan Journal of Medical Sciences 24: 209–212.

[50] Zeng XQ, Pan DD, Guo YX (2010) The probiotic properties of lactobacillus buchneri P2. J Appl Microbiol 108: 2059–2066. 19912431 10.1111/j.1365-2672.2009.04608.x

[51] Williams BL, Hornig M, Parekh T, Lipkin WI (2012) Application of novel PCR-based methods for detection, quantitation, and phylogenetic characterization of sutterella species in intestinal biopsy samples from children with autism and gastrointestinal disturbances. Mbio 3: e00261. 22233678 10.1128/mBio.00261-11 PMC3252763

[52] Cheng J, Kalliomäki M, Heilig HG, Palva A, Lähteenoja H, et al (2013) Duodenal microbiota composition and mucosal homeostasis in pediatric celiac disease. BMC gastroenterology 13: 113. 23844808 10.1186/1471-230X-13-113 PMC3716955

[53] Whitford M, Yanke L, Forster R, Teather R (2001) Lachnobacterium bovis gen. nov., sp. nov., a novel bacterium isolated from the rumen and faeces of cattle. Int J Syst Evol Microbiol 51: 1977–1981. 11760937 10.1099/00207713-51-6-1977

