# Supplementary figures and images for: Gut Microbiota Signatures Predict Host and Microbiota Responses to Dietary Interventions in Obese Individuals
Source: PLoS One. 2014 Mar 6;9(3):e90702. doi: 10.1371/journal.pone.0090702 (PMC3946202; doi:10.1371/journal.pone.0090702)

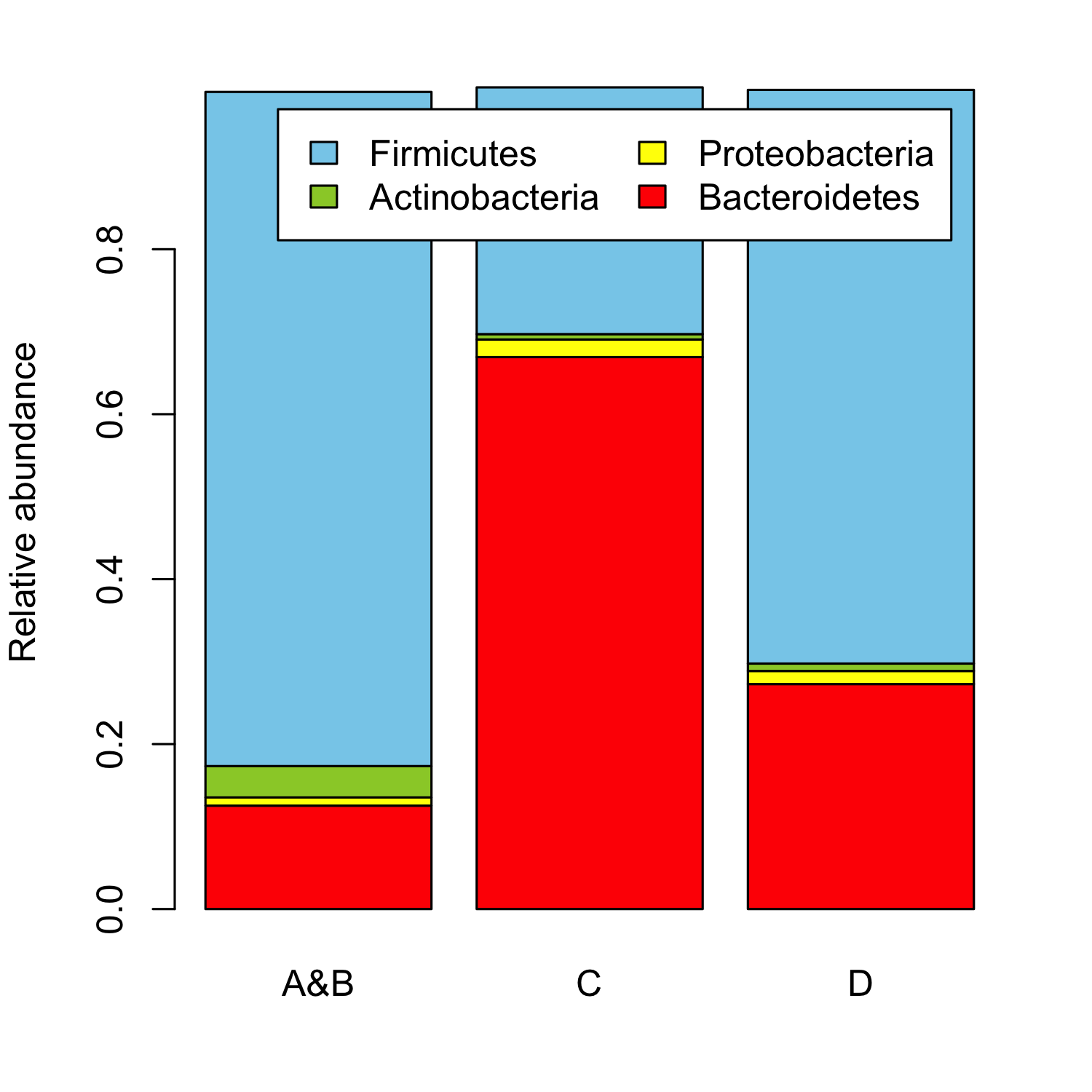

Supplement: Figure S3 — Relative abundances of the dominant phyla in the different studies (A–D) before data normalization. (TIFF) [file pone.0090702.s003.tiff]

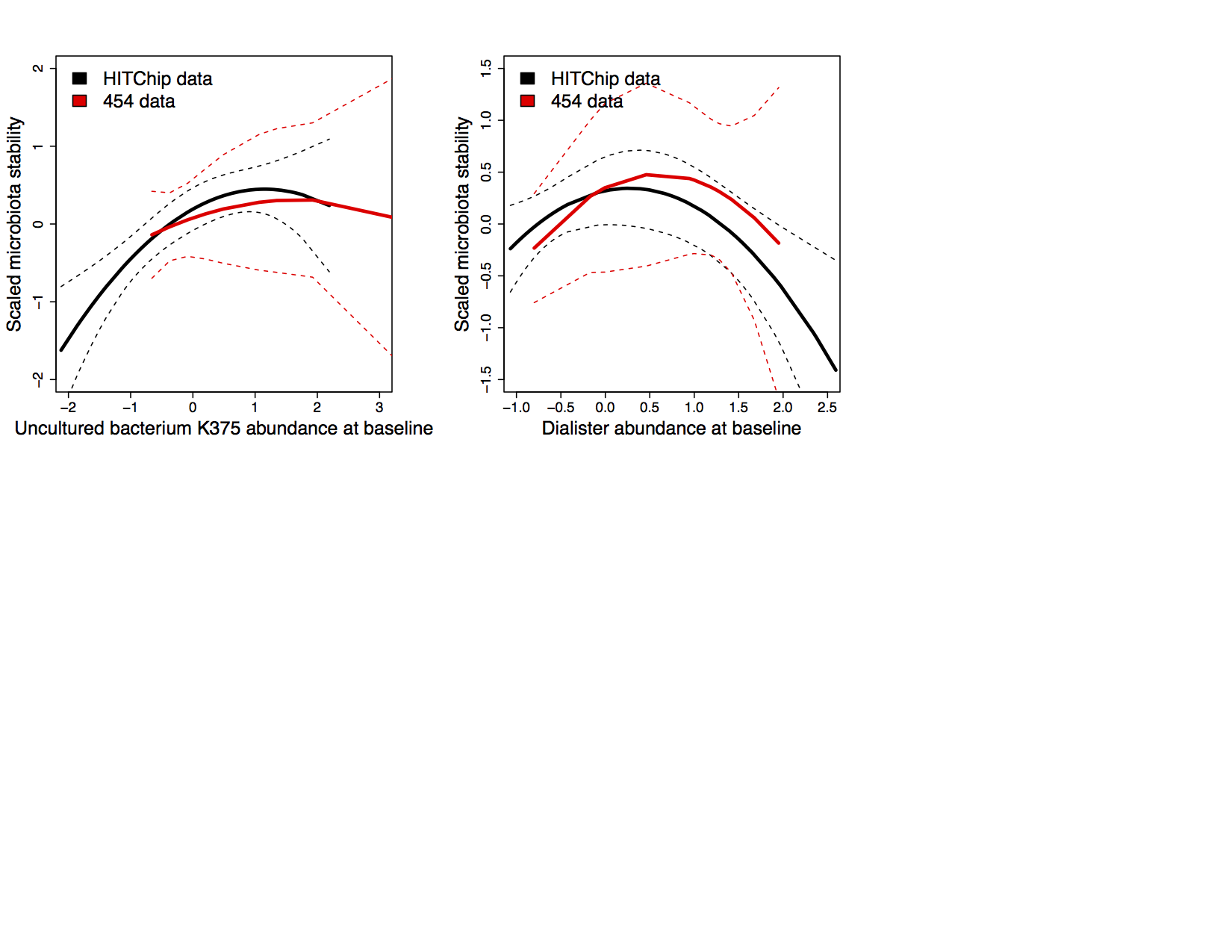

Supplement: Figure S8 — Relationship between the abundance of uncultured bacterium K375 and Dialister spp., with microbiota stability, measured with the HITChip in European studies and by 454 sequencing in an American study. The average abundances and stabilities were not comparable between platforms and were therefore scaled to mean = 0, and sd = 1. (TIFF) [file pone.0090702.s008.tiff]
