# Supplementary material for: Gut Microbiota Signatures Predict Host and Microbiota Responses to Dietary Interventions in Obese Individuals
Source: PLoS One. 2014 Mar 6;9(3):e90702. doi: 10.1371/journal.pone.0090702 (PMC3946202; doi:10.1371/journal.pone.0090702)
Supplement: Table S1 — Parameter estimates of the models for microbiota and host responses. The larger the estimate, the stronger the effect; negative values indicate a negative relationship and vice versa. The intercept is the estimated value of the response variable when all predictors are 0. (DOCX) [file pone.0090702.s009.docx]

Table S1. Parameter estimates of the models for microbiota and host responses. The larger the estimate, the stronger the effect; negative values indicate a negative relationship and *vice versa*. The intercept is the estimated value of the response variable when all predictors are 0.

| Parameter | Estimate | p |
| --- | --- | --- |
| Linear model for microbiota response |  |  |
| Intercept | -7.659 | 0.318 |
| *Eubacterium ruminantium* | 20.382 | <0.001 |
| *(Eubacterium ruminantium^2)* | -2.566 | <0.001 |
| *Clostridium sphenoides* | -0.174 | 0.049 |
| uncultured bacterium K305 | -10.241 | 0.025 |
| (uncultured bacterium K305^2) | 1.361 | 0.013 |
| *Lactococcus* sp 451 | -3.138 | 0.010 |
| uncultured bacterium K375 | -1.526 | <0.001 |
| *(Lactococcus* sp 451^2) | 0.309 | 0.057 |
| (uncultured bacterium K375^2) | 0.121 | <0.001 |
| *Oscillospira guillermondii* et rel | -0.088 | 0.432 |
| *Dialister + Uncultured Selenomonadaceae* | -0.518 | 0.707 |
| *Eubacterium ruminantium:(Dialister* + Uncultured *Selenomonadaceae)* | -2.697 | <0.001 |
| *(Eubacterium ruminantium^2):(Dialister* + Uncultured *Selenomonadaceae)* | 0.338 | <0.001 |
| *Clostridium sphenoides:(Dialister* + Uncultured *Selenomonadaceae)* | 0.034 | 0.033 |
| uncultured bacterium K305*:Oscillospira guillermondii et rel* | -0.072 | <0.001 |
| uncultured bacterium K305*:(Dialister + Uncultured Selenomonadaceae)* | 2.621 | 0.002 |
| (uncultured bacterium K305^2)*:(Dialister* + Uncultured *Selenomonadaceae)* | -0.320 | 0.002 |
| uncultured bacterium K375*:Oscillospira guillermondii et rel* | 0.126 | <0.001 |
| uncultured bacterium K375:*(Dialister* + Uncultured *Selenomonadaceae)* | 0.056 | 0.009 |
| *(Lactococcus* sp 451^2)*:(Dialister* + Uncultured *Selenomonadaceae)* | 0.015 | <0.001 |
| *(uncultured bacterium K375^2):Oscillospira guillermondii et rel* | -0.010 | <0.001 |
| (uncultured bacterium K375^2):(*Dialister* + Uncultured *Selenomonadaceae)* | -0.004 | 0.008 |
| Logistic model for microbiota response category |  |  |
| Intercept | 1552.993 | 0.001 |
| *E ruminantium + C felsineum* | -411.840 | 0.001 |
| *(E ruminantium + C felsineum)^2* | 27.231 | 0.001 |
| Logistic model for cholesterol response |  |  |
| Intercept | 22.274 | 0.994 |
| study A | 21.526 | 0.995 |
| study B | 20.343 | 0.995 |
| study C | 2.523 | 1.000 |
| *Clostridium sphenoides* | -11.066 | 0.020 |
| Linear model for HOMA response |  |  |
| Intercept | 367.808 | 0.020 |
| *Lactobacillus buchneri* | 0.230 | 0.943 |
| *(Lactobacillus buchneri^2)* | 0.922 | <0.001 |
| uncultured bacterium M615 | -215.019 | 0.011 |
| (uncultured bacterium M615^2) | 27.087 | 0.014 |
| bacterium adhufec335 | -1.562 | <0.001 |
| (bacterium adhufec335^2) | 0.138 | <0.001 |
| *Eubacterium biforme* | 16.313 | <0.001 |
| *(Eubacterium biforme^2)* | -1.413 | <0.001 |
| *Eubacterium contortum* | 8.472 | 0.008 |
| *Sutterella wadsworthia* | -85.006 | 0.012 |
| *Lactobacillus buchneri:Eubacterium contortum* | -2.026 | 0.013 |
| uncultured bacterium M615:*Sutterella wadsworthia* | 47.258 | 0.008 |
| (uncultured bacterium M615^2)*:Sutterella wadsworthia* | -5.965 | 0.011 |
| *Eubacterium biforme:Sutterella wadsworthia* | -2.988 | <0.001 |
| *(Eubacterium biforme^2):Sutterella wadsworthia* | 0.253 | 0.001 |
| Linear model for CRP response |  |  |
| Intercept | -0.535 | 0.996 |
| div Clostridium cluster XI | -24.768 | <0.001 |
| (div Clostridium cluster XI^2) | 0.637 | <0.001 |
| (uncultured bacterium D416+bacterium adhufec250) | -1.715 | <0.001 |
| ((bacterium adhufec250+uncultured bacterium D416)^2) | -0.002 | 0.882 |
| uncultured bacterium cadhufec32c10 | -7.175 | 0.165 |
| (uncultured bacterium cadhufec32c10^2) | -0.265 | 0.753 |
| *Lachnobacterium bovis et rel* | 71.164 | <0.001 |
| *(Lachnobacterium bovis et rel ^2)* | -4.376 | <0.001 |
| uncultured *Leuconostoc* sp LabS38 | -17.323 | 0.003 |
| *Clostridium ramosum et rel* | 80.584 | <0.001 |
| div Clostridium cluster XI:uncultured *Leuconostoc* sp LabS38 | 0.581 | 0.020 |
| div Clostridium cluster XI:*Clostridium ramosum* et rel | -1.316 | 0.001 |
| (uncultured bacterium D416+bacterium adhufec250):uncultured *Leuconostoc* sp LabS38 | 0.273 | <0.001 |
| ((bacterium adhufec250+uncultured bacterium D416)^2)*:Clostridium ramosum* et rel | 0.005 | 0.015 |
| (uncultured bacterium cadhufec32c10^2):*Clostridium ramosum* et rel | 0.208 | 0.078 |
| *Lachnobacterium bovis* et rel : *Clostridium ramosum* et rel | -13.382 | <0.001 |
| *(Lachnobacterium bovis* et rel ^2) : *Clostridium ramosum* et rel | 0.823 | <0.001 |
